# Craniopharyngioma in a 58-Year-Old Adult Male: A Case Report and Review of Literature

**DOI:** 10.7759/cureus.45493

**Published:** 2023-09-18

**Authors:** Jaswanthi Dogiparthi, Smaran S Teru, Thomas J Bonitz, Chris Buzas

**Affiliations:** 1 Medical Education, Lake Erie College of Osteopathic Medicine, Erie, USA; 2 Ophthalmology, Lake Erie College of Osteopathic Medicine, Erie, USA

**Keywords:** elevated intraocular pressure, atypical findings, suprasellar tumor, rathke's pouch, headache disorders, diabetes insipidus, neuro-endocrine disorder, visual field loss, afferent pupillary defect, recurrent craniopharyngioma

## Abstract

Craniopharyngiomas are benign epithelial tumors derived from the suprasellar region of the brain. The classical presentation of midline craniopharyngiomas includes bitemporal hemianopsia. However, atypical presentations can lead to diagnosis delays and challenges in managing associated visual and endocrine deficits. The persistence of visual deficits and tumor regrowth despite surgical intervention emphasizes the intricacies of craniopharyngioma management. This underscores the significance of timely diagnosis in patients with visual disturbances and hormonal imbalances related to mass effect. Here, we present a case of a unique and rare recurrent craniopharyngioma in a 58-year-old male, featuring progressive and atypical visual disturbances, along with the development of endocrine dysfunction following multiple tumor resections.

## Introduction

Craniopharyngiomas, derived from the suprasellar region of the brain, are benign epithelial tumors. They can be classified into two primary histological subtypes: adamantinomatous and papillary, affecting the pediatric and adult populations, respectively. Epidemiological studies indicate a bimodal age distribution, with the highest incidence observed in children aged 5-14 years and adults aged 50-74 years. The annual incidence of craniopharyngiomas, regardless of histological subtype, ranges from 0.5-2 cases per million, comprising approximately 1.2-4% of all intracranial tumors [1]. These tumors are typically confined to the sella turcica, a midline depression in the sphenoid bone housing the pituitary gland and situated below the optic chiasm [2]. Due to their location and size, craniopharyngiomas can present with variable signs and symptoms, including mass effect-related manifestations such as headaches, visual impairments, and hormonal disturbances [1]. In this report, we present a distinctive case of recurrent craniopharyngioma in a 58-year-old Caucasian male, characterized by progressive and atypical visual disturbances, as well as the development of endocrinopathy following multiple tumor resections.

## Case presentation

A 58-year-old Caucasian male with a medical history including benign craniopharyngioma, right optic nerve atrophy, headaches, type 2 non-insulin dependent diabetes mellitus, anxiety, depression, hypertension (HTN), obstructive sleep apnea (OSA), and presbyopia with corrective lenses, was referred to the ophthalmologist's office from endocrinology due to a new complaint of diabetes insipidus (DI).

During evaluation, the patient reported blurred vision in his right eye. Given his previous diagnosis of a surgically removed craniopharyngioma and similar symptoms two years prior, the patient expressed heightened concern. He also mentioned experiencing headaches originating from his left eye, which he attributed to compensatory straining caused by impaired vision in his right eye. The patient's current medication list included hydrocodone/acetaminophen, clonazepam, enalapril, sulindac, nifedipine, desmopressin, metoprolol, citalopram, levetiracetam, levothyroxine, prednisone, and pantoprazole. Surgical history included craniopharyngioma resection, while social and family history were non-contributory.

Physical examination revealed the patient's vision as 20/70 OD (oculus dexter/right eye), whereas his vision was 20/20 OS (oculus sinister/left eye). The right eye exhibited a relative afferent pupillary defect. The measurements for intraocular pressure were recorded as 12 mmHg in the right eye and 13 mmHg in the left eye. Ishihara testing revealed reduced color vision (2/11) in the right eye and normal color vision (11/11) in the left eye. Fundoscopic examination of the right optic nerve did not reveal any signs of edema with distinct margins but did show diffuse pallor, indicating optic nerve atrophy. The presence of a relative afferent pupillary defect, visual acuity decline, and color vision loss raised concerns for evolving optic nerve disease.

**Figure 1:**
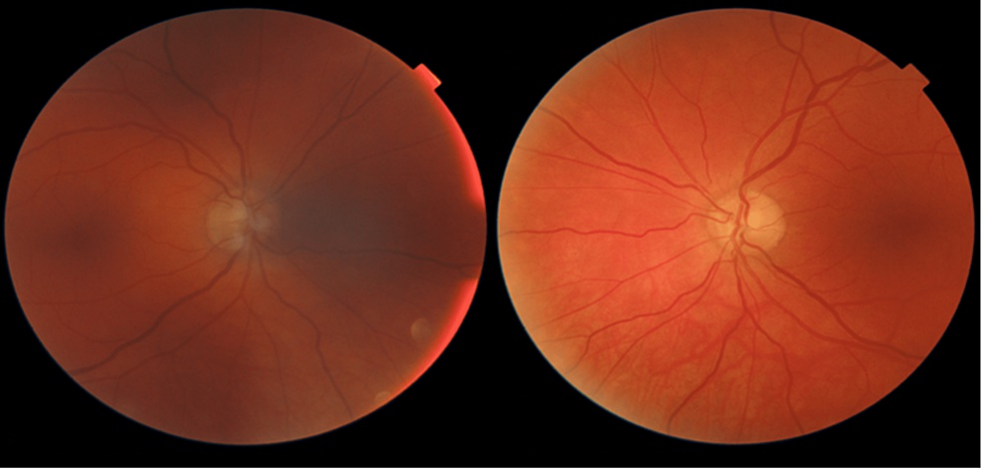
Fundoscopic examination showing moderate cupping, diffuse pallor, a dense superior and inferior nasal defect, and a central island OD (Left Image). Moderate cupping with a healthy rim, right superior temporal defect, and questionable junctional scotoma OS (Right Image). OD: oculus dexter (right eye); OS: oculus sinister (left eye)

**Figure 2:**
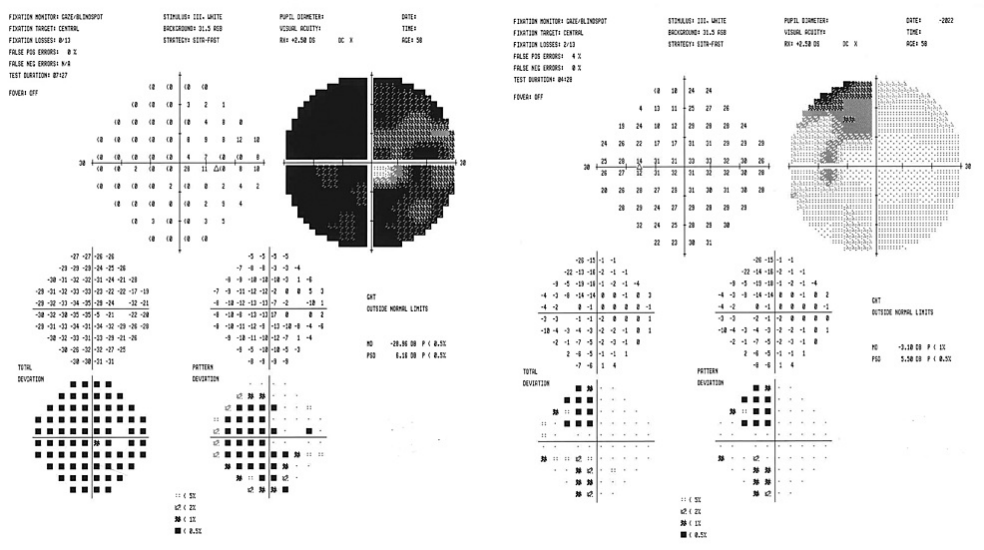
Visual field exam showing a significant visual field defect OD (Left Image). Superior temporal visual field defect OS (Right Image). OD: oculus dexter (right eye); OS: oculus sinister (left eye)

Fundoscopic examination (Figure 1) of the right eye revealed moderate cupping, diffuse pallor, a dense superior and inferior nasal visual field defect, and a central island. The left eye exhibited moderate cupping with a healthy rim, right superior temporal defect, and questionable junctional scotoma. Humphrey visual field exams (Figure 2) showed a significant visual field defect in the right eye, along with a superior temporal visual field defect in the left eye, consistent with a junctional scotoma. A brain MRI without contrast revealed a centrally located cystic suprasellar mass extending into the right midline. The mass measured 14.5 x 16.5 mm in two axial dimensions and 12 mm superior-inferiorly, displaying a relatively smooth wall and homogenous central signal intensity consistent with fluid. Ventriculomegaly remained unchanged from previous MRIs. Based on imaging, past medical history, and physical symptoms, there was a high suspicion of recurrent craniopharyngioma.

Neurosurgery successfully removed the intracranial mass, albeit with complications of cerebrospinal fluid (CSF) leak. An external ventricular drain (EVD) was inserted to address the CSF leak. During surgery, it was discovered that the mass was situated between the optic nerves and compressing the optic chiasm, contributing to the patient's visual changes. The histopathology report confirmed the extracted tumor as a recurrence of craniopharyngioma. Postoperatively, the patient developed hydrocephalus with an opening pressure of 22 mmHg, and 80 mL of CSF was drained over 24 hours via the EVD. Subsequently, a left parietal ventriculoperitoneal (VP) shunt was placed, effectively resolving the hydrocephalus. The patient reported significant vision loss and difficulties with vision testing in both eyes following surgery.

Six months later, the patient returned to the ophthalmologist's office due to persistent vision loss in the right eye and lack of peripheral vision in the left eye. He described his right eye as "fuzzy" and reported ongoing lacrimation. Postoperatively, he experienced balance difficulties and began using a cane for ambulation. The patient mentioned developing photophobia under certain lighting conditions and experiencing headaches if exposed for extended periods. He also mentioned that he is still experiencing intermittent floaters.

**Figure 3:**
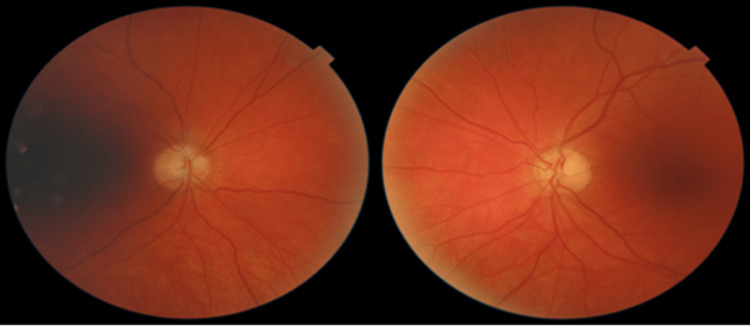
Fundoscopic examination showing diffuse pallor with small cupping OD (Left Image). Mild superior pallor with a small cup OS (Right Image). OD: oculus dexter (right eye); OS: oculus sinister (left eye)

**Figure 4:**
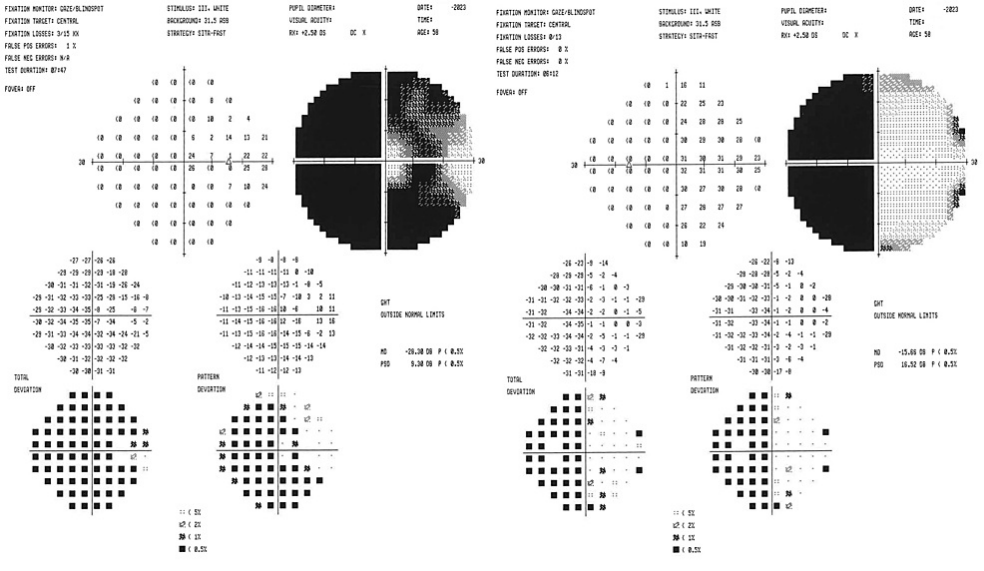
Repeat visual field examination showing significant visual field defect OD (Left Image). Temporal hemianopia OS (Right Image). OD: oculus dexter (right eye); OS: oculus sinister (left eye)

Upon examination, the patient's vision was measured as 20/30 in both eyes. The relative afferent pupillary defect persisted in the right eye, but the left eye was equal, round, and reactive to light and accommodation. The intraocular pressure measured 7 mmHg in the right eye and 10 mmHg in the left eye. Ishihara testing revealed reduced color vision (2/11) in the right eye and normal color vision (11/11) in the left eye. Fundoscopic examination indicated diffuse pallor with small cupping in the right eye, and mild superior pallor with a small cup in the left eye (Figure 3). Repeat Humphrey visual field examination confirmed previous findings, with a significant visual field defect in the right eye, with central island sparing. The superior temporal visual field defect (junctional scotoma) progressed to complete temporal vision loss, consistent with temporal hemianopia in the left eye (Figure 4). Optic atrophy persisted, with the right eye exhibiting significantly worse damage compared to the left eye. The patient was scheduled for follow-up brain MRIs every three months, with the possibility of extending intervals to six months if subsequent scans remained negative. As of the most recent update, the patient's vision has remained stable, and all MRIs conducted to date were stable.

## Discussion

Craniopharyngiomas, originating from Rathke's pouch, are rare benign tumors exhibiting a bimodal distribution, with a higher incidence in children [3]. Symptoms of craniopharyngiomas arise due to mass effect, resulting in headaches, visual deficits, cranial nerve disturbances, and hypothalamic or endocrine disorders caused by compression of the optic chiasm and pituitary gland [4]. However, misdiagnosing craniopharyngiomas in the adult population is common due to the presence of coexisting comorbid conditions like obesity or diabetes that may present similar symptoms.

Notably associated with craniopharyngiomas are pronounced visual changes. The optic chiasm, located approximately 10 mm superior to the sella turcica, serves as the convergence point for fibers from each optic nerve. These fibers intersect at the midline, joining with the non-decussating fibers of the contralateral optic nerve. The optic tracts, situated posteriorly to the optic chiasm, typically remain unaffected by craniopharyngiomas due to their location [2]. 

Lesions or compressions at the optic chiasm give rise to the pathognomonic pattern of visual loss known as bitemporal hemianopsia, characteristic of midline craniopharyngiomas. Decussating optic nerve fibers within the optic chiasm originate from the nasal or medial half of each retina, providing visual acuity to the temporal halves of the visual field [5]. Craniopharyngiomas, typically situated midline at the optic chiasm, induce visual deficits in the temporal fields bilaterally. Consequently, changes in vision are influenced by the tumor's size and location relative to the optic chiasm.

The presence of bitemporal hemianopsia should raise suspicion of a craniopharyngioma or other pituitary region tumors. This characteristic visual deficit facilitates the prompt diagnosis of this rare tumor, often presenting as an initial symptom due to optic chiasm compression [1]. However, the patient in this case did not exhibit bitemporal hemianopsia but instead experienced worsening vision in the right eye, followed by the left eye after tumor recurrence. During tumor resection on the first recurrence, it was observed that the tumor was midline, contrary to the patient's visual deficits. Worsening optic atrophy during multiple recurrences suggests pressure on the optic nerve rather than the optic chiasm. The patient's unique visual disturbances may be related to complications from diabetes insipidus, although this remains unconfirmed. The atypical visual presentations may have contributed to the delayed diagnosis, potentially explaining the persistence of visual deficits after tumor resection instead of the restoration of normal vision. Prolonged compression of the optic nerve by the tumor or iatrogenic stress can lead to optic nerve atrophy, as seen in this patient [6]. The patient's tumor likely compressed the right optic nerve, resulting in decreased right visual acuity in both medial and temporal fields, as well as left visual acuity deficits. Therefore, when patients present with signs of mass effect, including visual deficits, craniopharyngiomas should be considered and ruled out.

In addition to visual impairment, endocrinological changes are a common manifestation of craniopharyngiomas resulting from mass effect. Endocrine deficits commonly present as a sign of craniopharyngioma due to the compression of normal pituitary tissue located in the sella turcica. At the time of diagnosis, 40-87% of patients will present with at least one hormonal deficit [7]. Anterior pituitary deficiencies present more commonly as 40%, 85%, 25%, and 25% of patients have gonadotropin hormone-releasing hormone (GnRH), growth hormone (GH), adrenocorticotropic hormone (ACTH), or thyroid stimulating hormone (TSH) deficiencies, respectively [1]. Central diabetes insipidus (i.e., diabetes mellitus) is reported in approximately 20% of the patients with a pituitary hormone deficit secondary to craniopharyngioma, presenting as polyuria and polydipsia. The current patient was referred by the endocrinologist due to worsening of his diabetes insipidus along with worsening visual deficits. While comorbidities such as diabetes mellitus may present with very similar visual and endocrinological manifestations, craniopharyngiomas should be considered and ruled out.

The patient also presented with multiple recurrences of his craniopharyngioma despite surgical resection. Tumors can adhere to surrounding tissue, complicating the degree of resection and recurrence [1]. A systematic review and meta-analysis conducted in 2021 analyzed the rate of recurrence between the two histological subtypes of craniopharyngiomas: adenomatous (ACP) and papillary (PCP). The review included 974 patients from 13 studies and found that the ACP and PCP subtypes had recurrence rates of 26% and 14.1%, respectively. MRI without contrast analysis of the patient revealed a cystic suprasellar mass, highly suggestive of the ACP subtype [8]. Therefore, histological subtypes in a particular demographic may provide insight into relative rates of recurrence; however, this likely varies at an individual level. Introducing radiotherapy can provide therapeutic benefits to the patient by decreasing the risk of recurrence, but further research is needed to support this, as radiotherapy is currently being used as an adjunct to neurosurgery [2].

The patient also noted worsening visual presentation despite repeated surgical intervention. The patient's visual acuity decreased bilaterally, and optic atrophy was predominantly seen in the right optic nerve (and slightly in the left upon the most recent follow-up). This phenomenon conflicted with the results of a cohort study of 52 patients conducted between April 2002 and June 2011 at the University of Pittsburgh Medical Center, which assessed the visual outcomes after an endoscopic endonasal approach (EEA) [9]. Among the 42 patients in this study with quantifiable visual field defects on Humphrey or Goldmann perimetry, 37 patients documented improvements in their visual fields, while 17 patients experienced normalized visual fields between both eyes postoperatively. However, approximately one-third of the patients showed vision deterioration over years of long-term visual outcomes. In the current report, the patient initially had persistent right visual deficits, and after his second documented resection, he reported worsening vision in his right eye, with new deficits appreciated in his left eye. While many other factors likely contribute to the recurrence and poor visual prognosis despite surgical intervention, an appropriate and timely diagnosis of craniopharyngiomas should be made for better patient outcomes.

## Conclusions

This case report highlights a unique presentation of recurrent craniopharyngioma in a 58-year-old male. It is characterized by progressive and atypical visual disturbances, as well as the development of endocrinopathy following multiple tumor resections. While bitemporal hemianopsia is a hallmark sign of midline craniopharyngiomas, atypical presentations, like the one observed in this case, can lead to delayed diagnosis and create challenges in managing the associated visual and endocrine deficits. The patient's unique clinical course underscores the complexities of craniopharyngioma management. Timely diagnosis and consideration of craniopharyngiomas in patients presenting with mass effect-related visual deficits and endocrine abnormalities remain crucial for optimal patient outcomes. Further research and understanding of the underlying mechanisms contributing to atypical presentations and recurrence rates of different histological subtypes are essential for improving the management and therapeutic approaches for craniopharyngiomas.
